# GenSensor Suite: A Web-Based Tool for the Analysis of Gene and Protein Interactions, Pathways, and Regulation

**DOI:** 10.1155/2011/271563

**Published:** 2011-12-13

**Authors:** Mark Gosink, Sawsan Khuri, Camilo Valdes, Zhijie Jiang, Nicholas F. Tsinoremas

**Affiliations:** ^1^Investigative Toxicology, Pfizer Inc., Kalamazoo, MI 49001, USA; ^2^Center for Computational Science, University of Miami, Miami, FI 33146, USA; ^3^The Dr. John T. Macdonald Foundation Department of Human Genetics, Miller School of Medicine, University of Miami, Miami, FL 33136, USA; ^4^Department of Medicine, Miller School of Medicine, University of Miami, Miami, FL 33136, USA

## Abstract

The GenSensor Suite consists of four web tools for elucidating relationships among genes and proteins. GenPath results show which biochemical, regulatory, or other gene set categories are over- or under-represented in an input list compared to a background list. All common gene sets are available for searching in GenPath, plus some specialized sets. Users can add custom background lists. GenInteract builds an interaction gene list from a single gene input and then analyzes this in GenPath. GenPubMed uses a PubMed query to identify a list of PubMed IDs, from which a gene list is extracted and queried in GenPath. GenViewer allows the user to query one gene set against another in GenPath. GenPath results are presented with relevant *P*- and *q*-values in an uncluttered, fully linked, and integrated table. Users can easily copy this table and paste it directly into a spreadsheet or document.

## 1. Introduction

Transcriptome analysis and other high-throughput analyses of gene expression and proteomics technologies typically yield large lists of differentially expressed genes or proteins. These lists remain impenetrable without a second tier of analysis that subdivides them into functional categories ready for interpretation. Several methods have been developed to do this, the most widely used of which is Gene Set Analysis (GSA) [1, 2]. In GSA, the list of differentially expressed genes, called the test list, is compared to gene sets, which are lists of genes that have been classified into categories and deposited into accessible databases. Gene sets which are significantly over- or under-represented by genes in the test list are reported, with relevant statistical significance. This gives the researcher a starting point for functional genomics analysis and a more in-depth look at the gene expression profiles of the experiment.

The GSA approach was first applied to gene sets created from Gene Ontology (GO) classifications and was implemented in a large number of tools [3]. Recently the approach was extended to include other gene sets such as KEGG pathways, chromosomal locations, cis-regulatory elements, and indeed any gene set category relevant to the experiment at hand. It is beyond the scope of this paper to list all the tools that have been developed in recent years for comparing test lists against gene sets, it suffices to state that 68 were recently reviewed [4], and that the most commonly used of these tools are DAVID [5, 6] and GSEA [7]. This paper is to introduce the GenSensor Suite as a customizable, user-friendly tool with additional functionalities not often found in a standard GSA tool.

From the point of view of a wet-lab researcher, the number one priority is to obtain an accurate answer as quickly as possible from a freely available tool that is ultra-user-friendly. Ideally this tool would also allow literature searching and pathway analysis. No such tool currently exists, and this was the motivation behind the GenSensor Suite.

Designed specifically for end user biologists, the GenSensor Suite was developed to meet the needs for a statistically valid over-/underrepresentation analysis tool that is easy and quick to use, allows searching of several different gene sets in one standardized format, allows literature searching and investigation of interaction data, and, in particular, allows users to input their own reference data set. The suite is made up of four tools, as detailed below.

## 2. Methods

### 2.1. Implementation

The GenSensor Suite is implemented as a collection of common gateway interface (CGI) scripts that use Perl and JavaScript to handle HTTP requests and responses. The graphical user interface (GUI) is implemented using HTML, CSS style sheets, and JavaScript, and it is delivered to client systems using the Apache 2 HTTP Server. Performance has been tested in FireFox, Safari, Google Chrome, and Internet Explorer versions 7 and above, using PC as well as Apple environments. Over 1000 genes were analyzed in 1–3 seconds on fast (~25 Mbps) and slower (~2.5 Mbps) internet connections.

Some GSS functionality requires that JavaScript is enabled in the browser. All background gene lists are stored as text files of Entrez gene identifiers. New background lists can be created from microarray chip annotation files, and users can upload their own custom background list as a text file. The gene sets are species specific and stored as flat files of related gene categories. They consist of a one word category identifier, a short description of the category, and a list of Entrez Gene Identifiers belonging to that category. Any one gene can appear in multiple lists. Users can create or update gene sets through normal text file procedures.

After evidence in Rivals et al. [8], we use the fisher.test package, which is part of the default “stats” R package [9] set. We then use the “*q*-value” library from the Bioconductor set of packages for multiple testing correction of statistical significance [10–12]. By making use of the R language and libraries, GSS can perform fast calculations and take advantage of the Comprehensive R Archive Network (http://cran.r-project.org/) to integrate new or updated algorithms into the suite.

GSS is available for download by email request through the GNU General Public License (GPL) and is distributed as a compressed archive containing installation instructions and maintenance details. GSS is freely available at http://bio.ccs.miami.edu/GSS/. Users can deploy the software in Apple Mac OS X, Linux, and/or Microsoft Windows servers that support the Apache HTTP Server, the MySQL database, Perl, and R.

### 2.2. Gene Sets

Our predefined gene set categories were chosen to reflect the information that is most sought after in a typical biomedical or pharmaceutical sciences laboratory.  Table 1 shows a complete list of gene sets and how they were generated. These include gene sets of all KEGG pathways [14] and GO Terms [13] and custom-generated gene sets for transcription factor predicted targets, miRNA predicted targets, IPI Protein Subcellular Locations, disease-related and drug-related cocited genes (both based on MESH annotations), and a gene set of tissue specific genes based on the SymAtlas [15]. These gene sets are currently available for *Drosophila*, human, mouse, and rat.

The gene sets are updated regularly, and we can add new gene sets, such as for yeast or other model organisms, upon request via Contact Us. Gene sets and background list are in Entrez ID format [16]. Both gene ID and gene symbols are accepted in the input gene list; the tools use NCBI's latest *gene_info* file to map gene symbols to gene IDs for the analysis.

## 3. Functionality

The GenSensor Suite includes four tools for the analysis of large lists of genes or proteins. Each tool plus its manual is accessible with one click, and all user interfaces are clean and uncluttered.

### 3.1. GenPath

**Figure 1:**
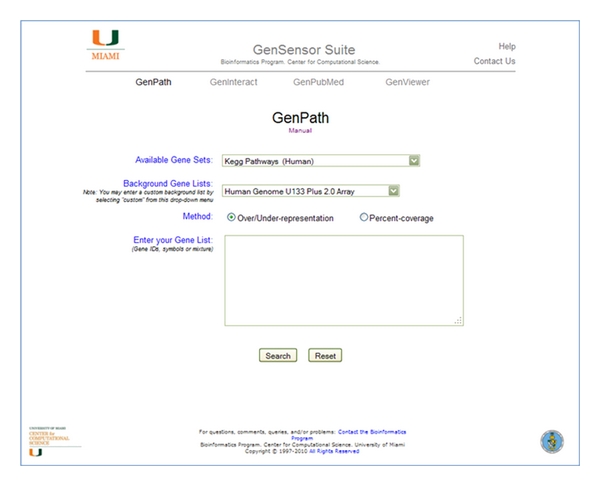
The homepage of the GenSensor Suite opens directly to the GenPath query page. All available gene sets are found in the first pull-down menu. Background gene lists and the ability to input your own custom background list are available from the second pull-down menu. After selecting the analytical method, you can paste your gene list into the input box and click on Search.

GenPath runs a test list of genes through a GSA method which queries a selected gene set, statistically compares the results with those using a background gene list, and presents the final results. Users choose the gene set from the first drop-down menu on the GenPath homepage (Figure 1) and can run GenPath as many times as they need on the same test list to gain a full perspective of their data. Users then specify the background list from which the test list was generated. In the case of a microarray experiment, the background is the set of probes on the array chip. The input list can be as Entrez Gene ID, gene symbol, or a mixture of both.

A particular feature of the GenSensor Suite is that users can input their own custom background lists according to their own specific line of research, such as a list of known disease-related genes, secreted proteins, drug targets, or toxicology gene lists. Select the “Input a custom gene list” option to open a new text box in which you can paste your reference list (this feature is currently available in Internet Explorer, Google Chrome, and Safari). This feature is particularly useful in pharmacogenomics and transcriptomics, where the background is essentially the mRNA data set of the species under study.

**Figure 2:**
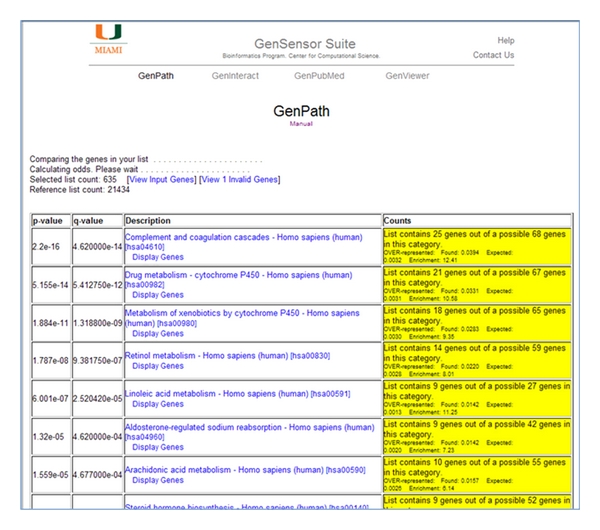
The GenPath results table. The results show all the gene sets found to be over- (yellow Counts) or under- (blue Counts) represented in your input gene list. They are displayed in order of significance, most significant being at the top. The example shown is of KEGG pathways, showing that the most significant pathway, with a *P* value of 2.2*e* − 16 and a *q*-value of 4.62*e* − 14, is the Complement and coagulation cascades pathway, where 25 genes from my list are found among the 68 genes connected with this pathway.* Data was generated using human kidney versus liver tissue microarray data from Marioni et al. *[17]*. We took genes that were ranked as “present” in all three replicates of both tissues, and that had a fold change differential of greater than 2. The resulting 636 genes were used as our test list. *

The user can run a GSA over- or under representation algorithm, or a percentage cover algorithm. The number of genes found in the test list which are over- or under represented in a gene set as compared to the background list are analyzed using Fisher's exact test. A multiple testing correction is applied using the *q*-value R library which implements Storey's *q*-value correction [11, 12]. The GenPath output is a table comprising the resulting list of gene sets, ranked by statistical significance down to a *P* value of ≤0.05 (Figure 2). Due to the possibility that our multitesting correction method might overcorrect, we display *q*-value results >0.05, in case the pathway(s) that the user expects to see falls just below the statistical threshold. The number of genes in the test list that are found in the gene set is given, along with a link to that list of genes and a link to the relevant database. Color coding of the cells in the right-hand column indicates categories which are overrepresented (yellow) or underrepresented (blue). The user is free to copy the table as it is and paste it into a spreadsheet or other document for further visualization and integration with other results.

### 3.2. GenInteract

**Figure 3:**
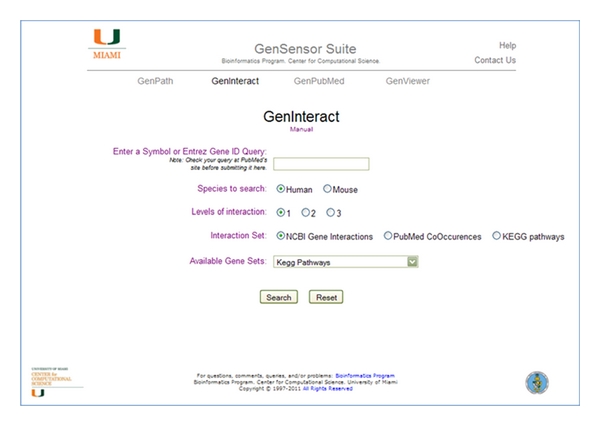
The GenInteract query page. In GenInteract, you start with a single gene ID or symbol, find genes that have been found to interact or be cocited with it, at a depth level of your choosing, and analyze the complete list directly in GenPath.

In GenInteract, users enter a single gene symbol or Entrez ID, choose the species in question, and select the depth of interactions to query (1, 2, or 3), and the source of interaction data (Figure 3). There are three interaction data sets to choose from: the NCBI Gene Interaction set is derived from the Interactions data at NCBI's Entrez Gene database [16]; the PubMed Co-Occurrences data set is based on the gene-to-PubMed data in NCBI's Entrez Gene database, whereby two genes are considered to form an interacting pair if they are both linked to five independent publications; the KEGG pathways data set is derived from the KEGG Markup Language (KGML) annotated pathways [14], and two genes are considered paired if one or more KGML pathway files describe a relation (of any description) between the two genes. The species and interaction data source that are chosen are used to build a list of all genes interacting directly with the query gene. If a depth of interaction greater than one is selected, the genes in this first resulting list are used in an iterative fashion to identify their respective interacting genes and produce the second level interactions gene list. The final list of all interacting genes, together with the background list of all genes which have interaction data (i.e., all genes in any KEGG pathway for KEGG interactions), are passed to GenPath, and the resulting pathway data is provided. Just above the results table is a link to the gene list that was generated, users can copy this and paste it into a text file for further analysis.

It must be clarified that this tool makes no inference as to the type of interaction that two genes might have between them. Whether an interaction is functional or simply a result of cocitation would depend on the source of interaction data that the user chooses.

### 3.3. GenPubMed

GenPubMed generates a co-citation gene list using a PubMed query and inputs that into GenPath. Users can enter Boolean searches and search field tags to generate complex PubMed queries. Unlike other enrichment tools capable of literature searches, such as Martini [18] and CoPub [19], GenPubMed maps the results of literature searches to genes rather than a dictionary of key words. This gene-centric approach means that GenPubMed can be used to search the same gene sets as used in other gene set analysis applications. Tools such as Martini use a literature search or gene list to pull back a list of PubMed IDs which are mapped to dictionaries of key words and terms. These terms are then analyzed for statistical overrepresentation. Our tool is designed to assist in literature searches and in data mining information and gene lists from PubMed. The user enters a text query either as a gene ID or as gene symbol or in standard PubMed syntax and chooses the organism to work with. We currently have the ability to query human and mouse data, and are working towards including other species. The query is passed to NCBI's PubMed site and a list of all matching PubMed IDs is generated. Then, a list of genes with links to these PubMed IDs is extracted from NCBI's Gene database. This gene list is the test list, the list of all genes with PubMed links is considered the background list, and both are passed to GenPath for analysis using the selected gene set category.

### 3.4. GenViewer

GenViewer allows individual gene sets to be browsed and selected. The list of genes from any gene set can then be queried through different gene sets in GenPath. For example, individual GO terms within a particular gene set in GO can be analyzed against the KEGG pathways. This is particularly useful when an unfamiliar GO term, or KEGG pathway, is given as a result in GenPath. The user might explore that category further through GenViewer and possibly find links with other pathways that may also be on the GenPath listing.

## 4. Conclusions

Over the past few years there has been a tendency in the biomedical research community to refer to these expanded GSA analyses as pathway analysis tools and include them in more of a system biology approach to gene expression analysis. In this context, a few applications have been developed that use one or other GSA method as one component in a pipeline of protocols that together allow some functional interpretation of the experimental results. Among the most popular of these tools are DAVID [5] and GSEA [7].

DAVID provides an easy-to-use application for the analysis of user-selected gene lists. Like our GSS tool, results are organized into tables of statistically significant gene sets. Unlike our tool, DAVID compares user lists to all gene set categories (i.e., GO terms, KEGG pathways, etc.) simultaneously with the resulting significant gene sets organized into functionally related “Annotation Clusters” which can allow the user to identify key biological features in such large result reports. A similar “clustering” of the related gene sets can be carried out in GSS using the GenViewer tool; however each gene set (i.e., for a single pathway) would have to be performed separately. DAVID is among the fastest GSA tools because all its statistical analyses are encoded into the java application. While we have sacrificed some speed for the flexibility in using external R calls, results from GSS are typically reported well within thirty seconds. Our tool also has several major features not available in DAVID, such as the ability to build a network of related genes using GenInteract and then analyzing for significant gene sets in a single step. Users of DAVID would have to build their list of interacting genes (and the appropriate background list) by hand. Additionally, GenPubMed takes advantage of the sophisticated Boolean queries available within NCBI's PubMed interface [20]. Utilizing GenPubMed, the user can get an overview of the key pathways or functional activities within an entire corpus of literature with a single click of the mouse.

GSEA offers a completely different method for analyzing gene set data albeit at the loss of some flexibility in the types of gene sets which can be analyzed. GSEA uses a nonparametric algorithm to compare ranked lists. This approach is extremely useful for analyzing gene data from one or more treatments where the rank order changes and then comparing those changed genes to gene sets. GSEA excels at comparison of treatment-induced gene changes to genesignatures built from previous experiments such as used in conjunction with the Connectivity Map [21]. However, this approach cannot be used to analyze gene lists where gene ranking is not applicable. Therefore, the majority of functions that are available in our GenInteract, GenPubMed, and GenViewer tools are not possible in GSEA.

In parallel, there have been several algorithms that take the information gained from GSA analysis and add a data-driven approach by building an interaction network within the reduced gene list of interest. Alongside this, a number of network visualization tools have been made available. The sum total of all these tools is impressive; however, the crucial point remains that the laboratory researcher often has very limited time available to learn, use appropriately, and get the best out of the plethora of sophisticated tools that are available. The GenSensor suite is an intuitive, integrated, accurate set of tools that a nonbioinformatics specialist can use with ease and confidence.

## Figures and Tables

**Table 1:** List of the gene sets available at the GenSensor Suite, and how they were generated. All gene sets are species-specific, and are updated regularly by utilizing the gene set construction scripts for a given gene set to parse the new updated annotations, and then replacing the old version with the new gene set.

Gene set	Description of contents
Gene ontology terms	Gene sets represent gene ontology categories. Each GO gene set contains genes annotated with the indicated term or any subterms [13]. *Details: gene ontologies were extracted from the gene2go file from NCBI's gene database. The GO terms and their relationships were extracted from the Ontologies file from the Gene Ontology web-site. Gene sets were created for genes matching each GO term. Matched genes also include matches to any GO subterm *

Kegg pathways	Gene sets represent the biosynthetic and regulatory pathways from the KEGG database [14]. *Details: pathway “.html” files were downloaded from KEGG. Entrez gene identifiers were extracted from the pathways imagemap annotations. Gene IDs from each extraction were made into separate gene sets. *

IPI protein subcellular locations	Gene sets are collections of genes whose proteins are annotated with particular subcellular locations. *Details: the UniProt formatted protein file was downloaded from the International Protein Index (IPI) at the EBI. Subcellular location terms were extracted from the “SUBCELLULAR LOCATION:” subsection of the Comment lines (CC) in the protein annotations. Unigene identifiers in the annotations were converted to Entrez gene identifiers using the “gene2unigene” file provided at the NCBI *

IPI protein key words	Gene sets are collections of genes whose proteins are annotated with particular key words. *Details: the UniProt formatted protein files were downloaded from IPI at the EBI. Key words were extracted from the Keywords lines (KW) in the protein annotations. Unigene identifiers were converted to Entrez gene as above *

Disease-related publication genes (*human only*)	Gene sets are collections of genes which are referenced in PubMed publications which are related to the disease terms found at MESH. *Details: the MESH ontologies were downloaded from National Library of Medicine's Medical Subject Headings (MESH) website. The lowest level terms (leaf nodes) were submitted to the PubMed website to identify all PubMed IDs matching the term as a MESH major topic. The genes described in each publication were identified using the gene2pubmed file at the NCBI. If a gene from a publication was nonhuman, the human homolog was identified using the Homologene data from NCBI. Publications discussing more than 100 genes were excluded as these generally were nonspecific discussion of EST libraries or microarrays *

Drug-related publication genes (*human only*)	Gene sets are collections of genes which are referenced in PubMed publications which are related to the chemical and drug terms found at MESH. *Details as above *

miRNA targets	Gene sets represent collections of potential gene targets of particular microRNAs as predicted by the Sanger Institute. *Details: the miRNA predicted targets and the miRNA data files were downloaded from the Sanger Institute. The “external identifier” in the targets file was converted to Entrez gene identifiers using files provided by the NCBI *

TF binding sites	Gene sets represent collections of genes with particular transcription factor binding sites located within 1000 bp upstream of their transcription start site. TF binding site predictions were made using minimal false positive or minimal false-negative settings. *Details: genomic sequences for the regions 1000 bp upstream of all human/mouse RefSeq transcripts were obtained from download pages for each organism from the UCSC genome browser. TRANSFAC analysis was performed using BioBase's “Match” tool. The “TF Binding Sites (min. false pos.)” set are the genes identified using the minimal false-positives vertebrate profiles (from minFP_good102.prf). Analyses were also performed with the minimal false-negative vertebrate profiles (from minFN_good102.prf) *

Tissue specific genes	Gene sets represent collections of genes whose expression is predominately confined to a few tissues. *Details: gene expression and annotation data for mouse was downloaded from the Genomics Institute of the Novartis Research Foundation's website. The intensities across all tissues were summed for each probeset. Intensity values for each probeset in each tissue were compared to the summed intensity value. If the intensity in a particular tissue was ≥25% of the total, the gene for that probeset was added to a collection of genes specific for that tissue *
